# Graphene oxide inhibits cell migration and invasion by destroying actin cytoskeleton in cervical cancer cells

**DOI:** 10.18632/aging.103821

**Published:** 2020-09-14

**Authors:** Jing Wang, Ping Wang, Ying He, Xiaoli Liu, Sisi Wang, Chunxing Ma, Xiaofei Tian, Jing Wang, Xin Wu

**Affiliations:** 1 Department of Gynaecology and Obstetrics, The First Affiliated Hospital of Hebei North University, Zhangjiakou, Hebei Province, China; 2 Operating Room, The First Affiliated Hospital of Hebei North University, Zhangjiakou, Hebei Province, China; 3 Department of Gynecology and Obstetrics, Hebei Maternity Hospital, Shijiazhuang, Hebei Province, China; 4 Basic Medical College, Hebei North University, Zhangjiakou, Hebei Province, China; 5 Life Science Research Center, Hebei North University, Zhangjiakou, Hebei Province, China; 6 Department of Pathology, Hebei North University, Zhangjiakou, Hebei Province, China

**Keywords:** graphene oxide, cytotoxicity, tumor metastasis, actin cytoskeleton, cervical cancer

## Abstract

Objective: To investigate the antitumor effects of Graphene oxide (GO) on tumor invasion and metastasis in human cervical cancer Hela cells.

Results: GO significantly inhibited cell viability and the number of clones, promoted cell apoptosis, as well as suppressed cell migration and invasion, and destroyed the structure of actin cytoskeleton of Hela cells in a dose-dependent manner in. Moreover, the expression of metastasis-related proteins, including MMP2 and Cdc42, were significantly suppressed by the treatment of GO. And the expression of MMP3 was remarkably increased by Smad inhibitor and the protein levels of MMP3 and ICAM were elevated by the JNK inhibitor in GO-treated Hela cells.

Conclusion: GO exhibited inhibitory effects on cell migration and invasion possibly by destroying actin cytoskeleton in Hela cells, which is a potential component of the Smad and JNK signalling pathways.

Methods: GO was prepared and chracterized by UV visible light absorption spectroscopy and atomic force microscopy. Hela cells were treated with Go at different dose levels. Then, in vitro cytotoxicity of GO was evaluated by the MTT assay, colony-forming assay and cell apoptosis assay. The inhibitory effects of GO on tumor cell migration and invasion as well as actin cytoskeleton were explored using Hela cells.

## INTRODUCTION

As the second most common gynecological malignancy among women worldwide, uterine cervical cancer is usually caused by persistent infection of carcinogenic human papillomavirus (HPV) [1]. According to relevant statistics, approximately 570 000 newly diagnosed cervical cancer cases and 311 000 death caused by cervical cancer were reported worldwide in 2018 [2]. Early diagnosis and effective treatment are the methods to prevent disease progress in patients with cervical cancer [3]. Over the years, rapid progress have been made in the diagnosis and treatment of cervical cancer, which contributed to the decrease in the disease’s mortality and morbidity [4]. Unfortunately, despite the effective therapeutic options of surgery and radiotherapy for early-stage cervical cancers, poor prognosis remains a challenge in the treatment of metastatic cervical cancer [5]. Therefore, it is essential to unveil the mechanisms of cervical carcinogenesis and explore novel therapies to combat tumor aggressiveness.

Currently, nanomaterials have been reported to boast promising prospect in detection and treatment of various types of malignancies due to their varied targeted roles in many fields [6], including imaging [7], immunodetection [8], chemotherapy [9], radiotherapy [10] and immunotherapy [11]. Notably, graphene is a single-layer sheet-like and two-dimensional sp2 hybridized carbon atoms that are packed in a hexagonal honeycomb crystalline structure [12]. Graphene oxide (GO), as an oxygenated derivative of graphene, contains various active oxygen-containing groups on its surface, and is usually prepared by treating graphene with a strong acid or a strong oxidant [13]. It has come to the forefront of attention owing to its exceptional physical and chemical properties, including good thermal stability, excellent mechanical strength and high electronic conductivity [13]. Recently, its use in biological fields has been explored, including drug delivery, biomedicine, cancer diagnosis and photothermography [14]. Several studies have confirmed the antitumor effects of GO as targeting drug carrier [15–17]. Moreover, several studies have reported that GO may have inhibitory potentials on the migration and invasion of tumor cells [18, 19]. However, the effectiveness and the related mechanism of action of GO in metastatic cervical cancer has been rarely investigated.

In the current study, the cytotoxicity and inhibiting effects of GO on tumor invasion and metastasis were explored using human cervical cancer Hela cell line, and the potential mechanism and signaling pathways involved were also investigated.

## RESULTS

### Characterization of GO

**Figure 1:**
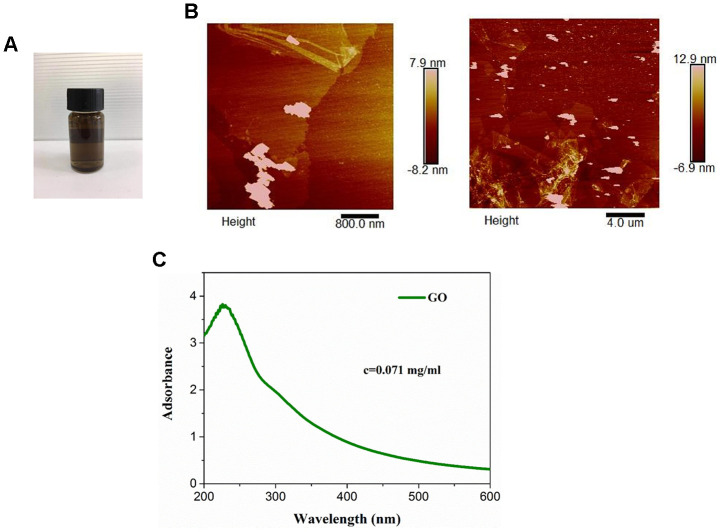
**Characterization of graphene oxide (GO).** (**A**) Appearance of commercial GO dispersion. (**B**) Ultraviolet visible absorption spectra of GO. (**C**) Representative images of GO using atomic force microscopy.

The color of commercial GO dispersion varied from yellow to brown (Figure 1A). The optical absorption spectra of GO showed absorption peaks at approximately (Figure 1B). As determined by atomic force microscopy, GO was 3-4 nm in thickness and 100-200 nm in diameter (Figure 1C), suggesting the formation of single or a few layers of GO nanosheets (Figure 1).

### Effect of GO on cytotoxicity in Hela cells

**Figure 2:**
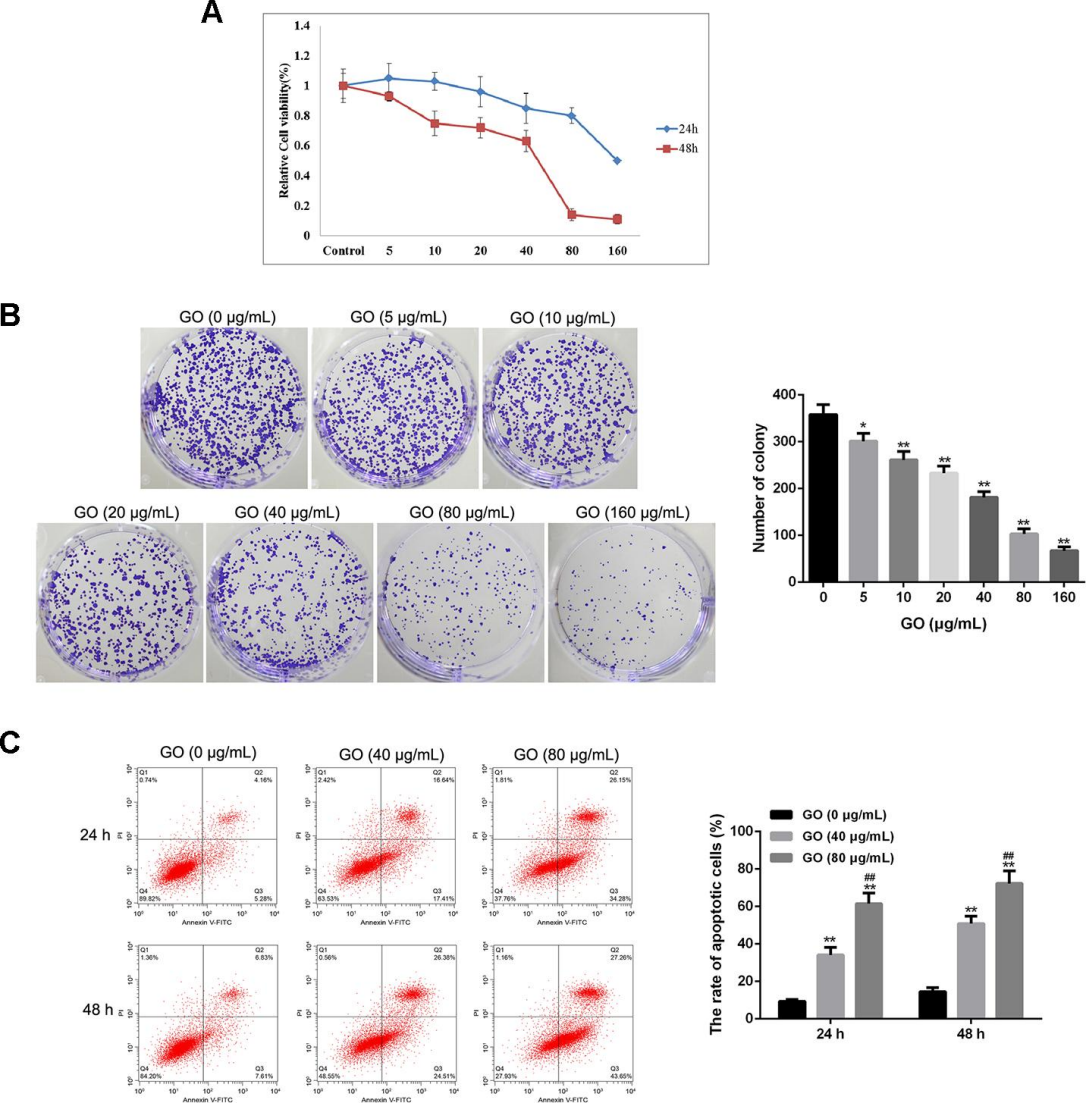
**Graphene oxide (GO) inhibits tumor growth in Hela cells.** (**A**) Cell viability of Hela cells treated with different doses of GO at 24h and 48 h by MTT assay. (**B**) Clone number of Hela cells treated with different doses of GO by colony-forming assay. (**C**) Cell apoptosis rate of Hela cells treated with different doses of GO at 24h and 48 h was calculated based on flow cytometry analysis. *P < 0.05, and **P < 0.01 vs control cells (0 μg/mL GO);f ##*P* < 0.01 vs cells treated with 40 μg/mL GO.

MTT assay showed that GO significantly inhibited cell viability in a dose-dependent manner both at 24 and 48 h, and the half maximal inhibitory concentration (IC_50_) was about 40 μg/mL (Figure 2A). Consistently, colony forming assay result also revealed that compared with control cells, the number of clones was obviously decreased in Hela cells treated with GO in a dose-dependent manner (Figure 2B). In addition, flow cytometry analysis found that the rate of apoptotic cells was remarkably increased after GO treatment in a dose-dependent manner both at 24 and 48 h in Hela cells compared with control cells (*p* < 0.01, Figure 2C). (Figure 2).

### Effect of GO on tumor metastasis in Hela cells

**Figure 3:**
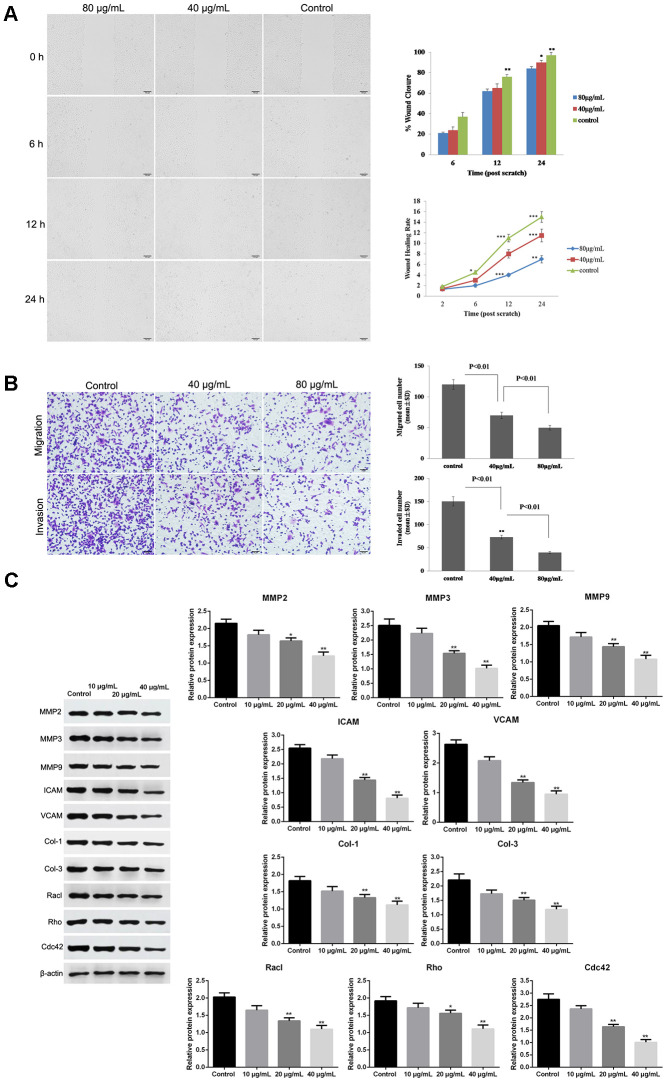
**Graphene oxide (GO) inhibited metastasis in Hela cells.** (**A**) The wound closure and wound healing rate of Hela cells treated with different doses of GO at 0, 6, 12 h and 24 h by wound healing assay. (**B**) Cell migration and migration rates in Hela cells treated with different doses of GO by Transwell assay. (**C**) Expression of metastasis-related proteins, including matrix metalloproteinase 2 (MMP2), MMP3, MMP9, intercellular adhesion molecule (ICAM), vascular cell adhesion molecule (VCAM), collagen type I (Col-1), Col-3, Racl, Rho and Cdc42, by western blotting. *P < 0.05, and **P < 0.01 vs control cells.

Wound healing assay showed that GO significantly decreased the wound closure and inhibited wound healing rate of Hela cells in dose- and time-dependent manner (*p* < 0.05, Figure 3A), suggesting a reduced migration tendency after GO treatment in Hela cells. Transwell assay also revealed that cell migration and invasion were dramatically suppressed in Hela cells treated with GO compared with control cells (*p* < 0.05, Figure 3B). Meanwhile, the expression of metastasis-related proteins, including MMP2, MMP3, MMP9, ICAM, VCAM, Col-1, Col-3, Racl, Rho and Cdc42, was detected by western blotting. The results demonstrated that GO treatment remarkably inhibited the protein levels of MMP2, MMP3, MMP9, ICAM, VCAM, Col-1, Col-3, Racl, Rho and Cdc42 in a dose-dependent manner compared with control Hela cells (*p* < 0.05, Figure 3C). (Figure 3)

### Effect of GO treatment on actin cytoskeleton in Hela cells

**Figure 4:**
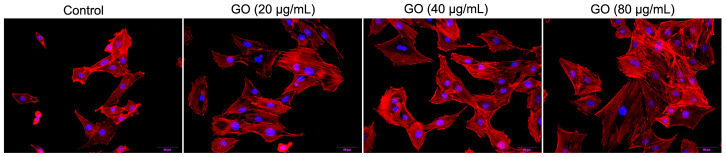
**Graphene oxide (GO) destroyed actin cytoskeleton of Hela cells.** Actin cytoskeleton of Hela cells treated with different doses of GO under confocal microscopy.

Due to the fact that actin cytoskeleton is essential for cell migration and invasion, the actin cytoskeleton of Hela cells was observed under a confocal microscope. As shown in Figure 4, in the cellular cytoplasm of control cells, actin filaments were found to be well arranged into thick bundles. In contrast, in Hela cells treated with GO, the structure of actin cytoskeleton was destroyed in a dose-dependent manner (Figure 4).

### Effect of GO on metastasis-related pathways in Hela cells

**Figure 5:**
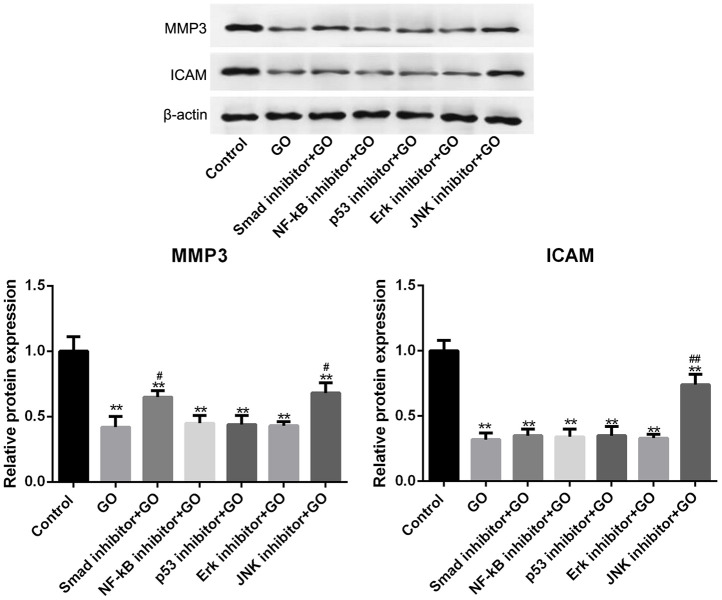
**Smad and JNK signaling pathways are associated with the inhibitory effect of graphene oxide (GO) on tumor metastasis in Hela cells.** The expression of MMP3 and ICAM in Hela cells co-treated with GO and Smad inhibitor, NF-kB inhibitor, p53 inhibitor, Erk inhibitor or JNK inhibitor by western blotting. **P < 0.01 vs control group, #*P* < 0.05, and ##*P* < 0.01 vs GO group.

Hela cells were co-treated with GO and several pathway inhibitors to identify the potential signaling pathways associated with the inhibitory effect of GO on tumor metastasis. The results revealed that compared with control cells, the protein levels of MMP3 and ICAM in Hela cells treated with GO were significantly inhibited (*p* < 0.01, Figure 5). Furthermore, MMP3 expression was obviously elevated by the addition of Smad inhibitor, and protein levels of MMP3 and ICAM in GO-treated Hela cells were remarkably increased by the addition of JNK inhibitor (*p* < 0.01, Figure 5). However, the addition of NF-kB inhibitor, p53 inhibitor and Erk inhibitor had no effect on the expression of MMP3 and ICAM (Figure 5).

## DISCUSSION

The current study revealed that GO significantly inhibited cell viability and the number of clones, promoted cell apoptosis, as well as suppressed cell migration and invasion in a dose-dependent manner in Hela cells. Moreover, GO treatment obviously damaged the structure of actin cytoskeleton of Hela cells. Furthermore, we found that GO might inhibit tumor metastasis by regulating Smad and JNK pathways.

GO has been widely used for various biological and biomedical fields due to its unique physiochemical properties, and its biocompatibility and safety are considered as the crucial factors in biomedical applications [20]. Several studies have evaluated the cytotoxicity of GO in *in vitro* experiments. Gurunathan S et al reported that GO could inhibit cell viability in a dose-dependent manner in MCF-7 cells, while GO with a dose level of > 60 μg/mL exerts significant cytotoxicity [21]. Zhou et al also evaluated the toxicity of GO in different types of malignancies, such as B16F10 mouse melanoma cells, PC3 human prostate cancer cells and MB-231 human breast cancer MDA- cells, and the results demonstrated the cytotoxicity of GO on these cancer cells and a dose-response relationship was identified [18]. In this study, we also evaluated the cytotoxicity effect of GO at different doses in Hela cells. The results showed that GO exhibited inhibitory effect on cell viability and the number of clones in a dose-dependent manner, while GO at a dose greater than 80 μg/mL was related with a lower cell viability at 48 h in Hela cells. Furthermore, cell apoptosis assay results revealed a low apoptotic rate at 40 μg/mL of GO and a high apoptotic rate at 60 (60??) μg/mL of GO. All these data suggested that GO with a low dose at 40 μg/mL had no apparent cytotoxic effect on Hela cells.

Tumor metastasis is a complex and multistep process involving formation of a new microvascular network, tumor cells invading the primary organ and detaching from the primary tumor to invade the surrounding tissues and infiltrate and proliferate in a new organ. High rate of tumor metastasis is considered a great challenge for cancer treatment. Interestingly, previous studies have shown that GO treatment were associated with slower rate of migration and invasion in several cancer cells [18, 19]. Thus, we further investigated the antitumor effect of GO on cell migration and invasion in Hela cells, and the results indicated that GO treatment suppressed migration and invasion of Hela cells. In addition, this study showed that GO could also damage the structure of actin cytoskeleton of Hela cells. Tumor cells have been known to undergo morphological and structural changes during the process of metastasis, and actin cytoskeleton play an important role in cancer progression by driving tumor cell invasion and migration [22–24]. Therefore, GO might suppress migration and invasion of Hela cells by destroying actin cytoskeleton.

Furthermore, we detected the expression of metastasis-related proteins, including MMP2, MMP3, MMP9, ICAM, VCAM, Col-1, Col-3, Racl, Rho and Cdc42, in Hela cells treated with and without GO. Of them, MMPs, MMP2, MMP3 and MMP9 could induce tumor cell invasion and migration by promoting the degradation of extracellular matrix, such as collagen [25]. ICAM and VCAM, both categorized as adhesion factors, could strengthen the adhesion between tumor cells and endothelial cells through specific binding to the receptors on the cells, thereby promoting tumor cell invasion and migration [26]. In addition, Rho protein family, consisting of Racl, Rho and Cdc42, can modulate cytoskeleton and cell adhesion, thereby playing an important role in promoting malignant transformation of cells as well as tumor invasion and metastasis [27]. Consistently, our study revealed that GO inhibited the expression of these metastasis-related proteins. In addition, we found that MMP3 expression was downregulated by Smad and JNK pathways, and ICAM expression was downregulated by JNK pathway. Thus, we speculated that the inhibiting effects of GO on cell migration and invasion might be closely associated with Smad and JNK pathways via downregulating MMP3 and ICAM.

## CONCLUSIONS

This study suggested that GO might inhibit cell migration and invasion through destroying actin cytoskeleton, which might be involved in the Smad and JNK signalling pathways.

## MATERIALS AND METHODS

### Preparation and characterization of GO

GO dispersion liquid was purchased from XFNANO Materials Tech Co. Ltd (Nanjing, Jiangsu, China). The ultraviolet (UV) visible spectra of GO was measured by UV visible light absorption spectroscopy (Biochrom, Cambridge, UK). The morphology and thickness of GO were evaluated by atomic force microscopy (AFM, JPK Instruments AG, Berlin, Germany).

### Cell culture and treatment

The human cervical cancer Hela cell line was obtained from Shanghai Obio Technology Co., Ltd, and then maintained in complete DMEM medium (Gibco, Carlsbad, CA, USA) under 37°C and 5% CO_2_. Hela cells were exposed to different dose of GO (5, 10, 20, 40, 80and 160 μg/mL) to evaluate the suppressing effects and cytotoxicity of GO on tumor metastasis. Moreover, to investigate the potential signaling pathways involved in the carcinogenesis, Hela cells were pretreated with Smad inhibitor, NF-kB inhibitor, p53 inhibitor, Erk inhibitor and JNK inhibitor (Sigma, St. Louis, MO, USA) for 30 min, and then exposed to 20 μg/mL GO for 24 h.

### MTT assay

Hela cells were grown in 96-well plates and incubated with GO at different dose levels. After conventional incubation for 24, and 48 h, each well was added with 10 μl of MTT (5 mg/mL, Sigma) to incubate for another 4 h. Then 100 μL of dimethyl sulfoxide (DMSO) was added. Finally, cell viability was evaluated using a microplate spectrophotometer to measure absorbance at 470 nm.

### Colony-forming assay

Hela cells were grown in 6-well plates at a density of 400 cells per well, and then were treated with GO at different dose levels for 14 days under standard culturing conditions. Then the cells were fixed with absolute methanol, and incubated with a crystal violet solution. Finally, the number of colonies was counted.

### Cell apoptosis assay

Hela cells were treated with 40 and 80 μg/mL GO, respectively, for 24 and 48 h. Trypsin was added to the plates to digest Hela cells, and then the cells were harvested. The cells were rinsed with PBS solution, and re-suspended in binding buffer. After the cells were incubated with FITC-Annexin V and propidium iodide (PI) for 15 min, the number of apoptotic cells was counted using a flow cytometer (BD, CA, USA).

### Wound healing assay

Hela cells were inoculated to 6-well plates. After growing to 60% of confluence, the cells were wounded by scratching with sterile plastic pipette tips vertically against the well, followed by culturing in DMEM without serum. Subsequently, the cells were treated with 40 and 80 μg/mL GO, respectively. The distance of the scratch was measured at 0, 6, 12 and 24 h using a light microscope (Olympus, Japan).

### Transwell assay

Tumor cell invasion and migration were elevated by Transwell chambers (Corning). Concretely, the lower compartment was filled with DMEM supplemented with with 10% FBS and GO (40 and 80 μg/mL). The Hela cells were grown in the upper compartment coated with Matrigel Matrix and cultured in medium free of serum for 24 h. Subsequently, the cells in the lower compartment was fixed and stained with 4,6-diamidino-2-phenylindole. And cell migration and invasion were evaluated using an inverted microscope (Olympus, Japan).

### Cytoskeleton staining

Hela cells were inoculated on cover slips, and then exposed to 0, 20, 40 and 80 μg/mL GO, respectively for 24 h. The cells were then fixed using 4% paraformaldehyde and permeabilized with 0.1% Triton X-100. After blockage with 2% bovine serum albumin, the cells were stained with 5-TAMRA- Phalloidin to visualize actin (red) and DAPI to identify nuclei (blue). Finally, cell images were obtained using laser scanning confocal microscope (Olympus, Japan).

### Western blotting analysis

Hela cells were treated with GO and/or pathway inhibitors for 24 h. The harvested cells were lysed with RIPA lysis buffer (Gibco) to extract proteins and the protein concentration was quantified using a commercial protein assay kit (Pierce, Rockford, IL, USA). The protein lysates were separated on PAGE gel and transferred onto PVDF membranes. Afterwards, the membranes were blocked and reacted with matrix metalloproteinase 2 (MMP2), MMP3, MMP9, intercellular adhesion molecule (ICAM), vascular cell adhesion molecule (VCAM), collagen type I (Col-1), Col-3, Racl, Rho, Cdc42 and β-actin primary antibody (1:300, Santa Cruz) overnight at 4°C. After incubation with secondary antibody (1:5000, Jackson, USA), the proteins were determined by enhanced chemiluminescence (ECL, Millipore, USA).

### Statistical analysis

Data was presented as mean ± SD and compared with one-way ANOVA followed by multivariate regression analysis using the SPSS software. *P* < 0.05 was considered statistically significant.
